# Bone Lengthening with a Motorized Intramedullary Nail in 34 Patients with Posttraumatic Limb Length Discrepancies

**DOI:** 10.3390/jcm10112393

**Published:** 2021-05-28

**Authors:** Maxime Teulières, Tristan Langlais, Jérôme Sales de Gauzy, Jan Duedal Rölfing, Franck Accadbled

**Affiliations:** 1 Department of Pediatric Orthopaedics, CHU de Toulouse, 31300 Toulouse, France; maxime.teulieres@gmail.com (M.T.); tristanlanglais@yahoo.fr (T.L.); salesdegauzy.j@chu-toulouse.fr (J.S.d.G.); 2 Children’s Orthopaedics and Reconstruction, Aarhus University Hospital, 8200 Aarhus, Denmark; jan.roelfing@rm.dk

**Keywords:** limb lengthening, bone lengthening, limb length discrepancy, LLD, MAD, mLDFA, MPTA, intramedullary lengthening nail, FITBONE, posttraumatic, fracture, complications

## Abstract

The Fitbone^®^ motorized nail system has been used to correct limb length discrepancies (LLD) for several years. This study focuses on its application in posttraumatic limb lengthening surgery, its outcome and challenges. Materials and methods: A prospective, single center study was conducted between 2010 and 2019 in patients treated with motorized lengthening nails. The inclusion criteria were symptomatic LLD of 20 mm or more. An imaging analysis was done using TraumaCad^®^ software (Brainlab AG, Munich, Germany) to compare frontal alignment angles and limb length discrepancy (LLD) on preoperative and latest follow-up radiographs of the lower limbs. Results: Thirty-four patients were included with a mean age of 28.8 ± 9.7 years, a mean follow-up of 27.8 ± 13 months and a mean hospital stay of 4.4 ± 1.7 days. The mean LLD was 44 ± 18 mm in 29 femoral and 32 ± 8 mm in 4 tibial cases, which was reduced to less than 10 mm in 25/34 (74%) patients. The mean healing index was 84.6 ± 62.5 days/cm for femurs and 92 ± 38.6 days/cm for tibias. The mean time to resume full weight-bearing without walking aids was 226 days ± 133. There was no significant difference between preoperative and final follow-up alignment angles and range of motion. The mechanical lateral distal femoral angle (mLDFA) was corrected in the subgroup of 10 LLD patients with varus deformity of the femur (preoperative 95.7° (±5.0) vs. postoperative 91.5° (±3.4), *p* = 0.008). According to Paley’s classification, there were 14 problems, 10 obstacles and 2 complications. Discussion: Six instances of locking screw pull out, often requiring reoperation, raise the question of whether a more systematic use of blocking screws that provide greater stability might be indicated. Lack of compliance can lead to poor outcomes, patient selection in posttraumatic LLD patients is therefore important. Conclusion: Limb lengthening with a motorized lengthening nail for posttraumatic LLD is a relatively safe and reliable procedure. Full patient compliance is crucial. In-depth knowledge of lengthening and deformity correction techniques is essential to prevent and manage complications.

## 1. Introduction

Lower extremity long bone fractures can cause limb length discrepancies (LLD) in case of unsuccessful reduction, fixation failure, or secondary displacement [1,2,3]. LLD after intramedullary nailing for femoral shaft fractures has been reported to affect up to 43% of patients, especially in complex and communitive fractures [4]. Growth plate injuries in skeletally immature individuals can also result in LLD [5,6].

LLD can cause limping, back pain, and secondary degenerative changes, i.e., osteoarthritis. Mild LLD, i.e., less than 2 cm, can most often be satisfactorily treated with a shoe raise. However, marked LLD of more than 2–2.5 cm may warrant limb lengthening surgery. Historically, external fixation was the preferred lengthening method [7]. However, technical breakthroughs have resulted in reliable motorized intramedullary lengthening nails, such as FITBONE^®^ (Orthofix, Lewisville, TX, USA) and PRECICE^®^ (NuVasive, Aliso Viejo, CA, USA) [7,8,9,10,11,12]. Intramedullary lengthening nails with full weightbearing capability allowing faster postoperative rehabilitation and simultaneous bilateral lengthenings are the frontier and logical next step of the evolution of these devices.

Compared with idiopathic LLD, posttraumatic patients are more likely to sustain complications when undergoing lengthening surgery due to pre-existing complicating factors, e.g., scare tissue, joint stiffness, dormant infection, skin issues, etc. [7,8]. This study investigates bone lengthening in posttraumatic patients with the FITBONE^®^ intramedullary nailing system at Toulouse University Hospital in France. Patient characteristics, lengthening modalities, radiographic analyses of the achieved lengthening and axial deformity correction, as well as complications are presented.

## 2. Materials and Methods

In this prospective, single center, single surgeon case series, 34 posttraumatic limb lengthening patients (femoral: 30, tibial: 4) were included from January 2010 until April 2019. Patient selection was based on the following inclusion criteria: patients with a symptomatic, posttraumatic LLD of 20 mm or more.

Causes of femoral LLD were 10 shaft fractures treated with intramedullary nails, 6 distal fractures (4 plates, 2 external fixators) and 3 proximal femoral fractures (2 intertrochanteric nails, 1 dynamic hip screw). Ten LLD cases were the result of trauma to the immature skeleton, among which 6 were due to physeal injuries. Causes of tibial LLD were 2 shaft fractures, 1 pilon fracture and 1 distal growth plate injury. One functional LLD was included in our cohort. The patient fell from a horse causing a Tile type C pelvic fracture with hemipelvic dislocation despite fracture fixation (Table 1).

In 15 cases, trauma was the result of a road traffic accident. Nine cases involved a motorcycle. Two gunshot injury patients were included in our cohort and 10/34 (29%) were open fractures. Gustilo Anderson classification of the open fractures was not possible, because multiple of the patients were referred several years after the initial trauma.

### 2.1. Surgical Technique

Preoperative planning followed the reverse planning method described by Baumgart using anteroposterior (AP), full-length long standing radiographs and the TraumaCad^®^ software (Brainlab AG, Munich, Germany) [13]. Accordingly, the osteotomy site and the position of the implant were mapped out to achieve the desired corrections. Implant insertion was either antegrade or retrograde for femoral lengthening and antegrade for tibial cases. The FITBONE^®^ TAA (Telescope Active Actuator) mechatronic implant also allowing was used in the 30 cases, while the SAA (Sliding Active Actuator) also allowing bone transport was used in 4 femoral cases. The TAA implant is available in 9, 11, and 13 mm diameters and is capable of lengthening up to 8 cm without recharging of the nail. In accordance with the instructions for use, no healthy, open growth plates were crossed by the lengthening nail.

Anteroposterior or mediolateral blocking screws were used if needed to correct deformities and/or further stabilize the implant [14,15,16].

Surgical procedure: The patient was positioned supine on a radiolucent operating table fitted with a plexiglass plate and a metal grid to assess the mechanical axis of the limb intraoperatively. Steinman pins positioned horizontally on each side of the osteotomy site (posterior femoral condyle and lesser trochanter for femoral lengthening) helped to prevent rotational malalignment or accurately correct rotational deformity if indicated. A percutaneous, multiple drill hole osteotomy was performed under fluoroscopic guidance before reaming in order to minimize the risk of fat embolism and to increase the amount of reaming debris at the osteotomy site [17]. According to the preoperative plan, Kirschner wires were inserted at standard entry points for ante- or retrograde nailing. This was used to insert a cannula for rigid intramedullary reaming under fluoroscopic guidance. After implantation, the nail was connected to a subcutaneously implanted receiver and the motor was tested under sterile conditions at the end of the procedure. A single shot nerve block was applied in all femoral, but none of the tibial cases.

### 2.2. Postoperative Follow-Up

**Figure 1:**
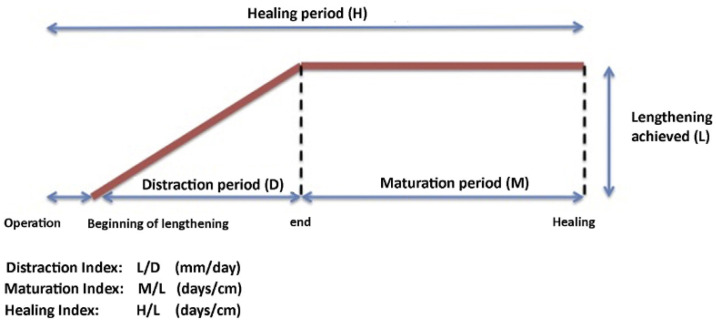
Lengthening phases and calculation of indices.

Lengthening started on postoperative day 3 for femurs and 5 for tibias. Additional days of latency were added in patients 40 years and older, with the presence of smoking and comorbidities. Physical therapy was started on postoperative day 1 to recover range of motion (ROM) and ambulation with a walking aid and limited weight-bearing up to 20 kg. The control receiver allowed 1 mm lengthening daily, divided into 3 sessions. During the distraction period (Figure 1) patients need close follow-up in the outpatient clinic, i.e., with 7–14 day intervals. Here, adequate bone formation was monitored with radiographs and range of motion was assessed at each visit. If necessary, the treatment plan could be adapted in order to prevent complications.

Compliance with daily physiotherapy is key in order to prevent loss of range of motion. If temporary loss of range of motion was noted during lengthening, daily physiotherapy sessions were increased. If we observe a loss of full extension or a temporary flexion contracture of the knee of more than 30 degrees, we paused the lengthening for 1 week in order to prevent subluxation and permanent joint contracture. The same protocol was applied if dorsiflexion of the ankle was reduced to 0 degrees or equinus. Moreover, we did not routinely prescribe orthoses, nor did we prophylactically lengthen tendons apart from the iliotibial band which was released in femoral lengthening of more than 3–4 cm.

During the maturation period, weight-bearing was resumed progressively. Full weightbearing was allowed once bone union was achieved, defined as at least 3 visible cortical bridges out of 4 cortices. All patients had a final clinical and radiographic follow-up six months after implant removal.

### 2.3. Lengthening Outcome Parameters

The outcomes measured were the achieved lengthening, duration of the distraction phase (days), distraction index (days/cm), healing index (days/cm), length of hospital stay (days) (Figure 1), lower limb ROM at the latest follow-up, time to resume full weight-bearing (days) and time to resume walking without aids (days).

The lengthening goal was achieved when it was within 5 mm of the initial plan. This corresponds to the LLD measurement error of long standing radiographs. Bone union was defined as at least 3 visible cortical bridges out of 4 in the regenerate on radiographic AP and lateral views. The pre- and postoperative mechanical axis deviation (MAD), mechanical lateral distal femoral angle (mLDFA) and medial proximal tibial angle (MPTA) were measured to assess alignment of the limb in the frontal plane. Intraoperative and postoperative complications were noted and categorized according to Paley’s classification: problem (grade 1), obstacle (grade 2) and complication (grade 3) and according to Black in grade I, II, IIIA, IIIB [18,19].

### 2.4. Statistical Analysis

Statistical analysis was performed using Student’s t test (paired data: before limb lengthening surgery and at latest follow-up). Data are presented a mean ± standard deviation. A *p*-value ≤ 0.05 was considered statistically significant.

## 3. Results

**Figure 2:**
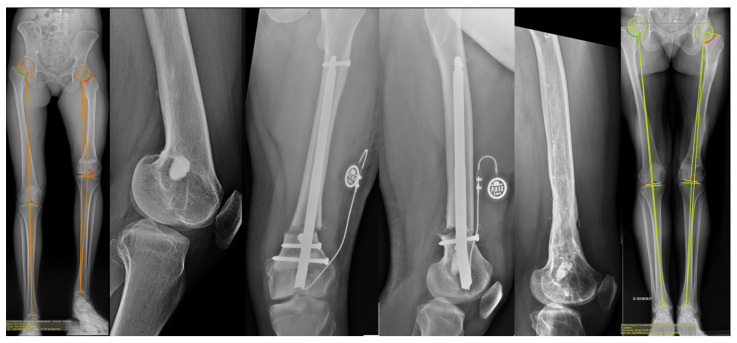
Radiographic images of a 26-year-old female with a 100 mm LLD associated with flexion and varus deformity due to growth plate injury at age 5 with subsequent attempted of Langenskiold procedure. 80 mm lengthening as well as correction in both the coronal and sagittal plane was performed with a retrograde FITBONE^®^ TAA nail.

A total of 34 patients with posttraumatic LLD and a mean age of 28.8 ± 9.7 years were included (Figure 2, Table 2). The mean LLD of 42.3 ± 17.2 mm, and a male/female-ratio of 2.7 (Table 2). The mean length of the hospital stay was 4.4 ± 1.7 days. Full weight-bearing and walking without aid resumed 139 ± 52 days and 226 ± 133 days after implantation, respectively.

The approach for femoral lengthening was retrograde in 21 segments and antegrade in nine segments (Figure 2). Blocking screws were applied for 12/34 (35%) lengthening surgeries (11 femurs and one tibia).

Mean lengthening was 37.5 ± 19 mm for the femur and 23.7 ± 7 mm for the tibia. A final LLD ≤ 5 mm was achieved in 12/34 patients, while 9/34 patients had a final LLD of more than 10 mm. Distraction stops were due to the development of ankle equinus, knee flexion contracture, infection of the surgical site, or interruption decided by the patient.

Mean distraction time was 60 ± 27 days and the mean distraction index was 0.59 ± 0.16 mm/days. The mean healing index was 84.6 ± 62.5 days/cm for the femur and 92.8 ± 38.6 days/cm for the tibia.

Implant removal was performed at mean 19.7 ± 7.7 months after implantation. The mean follow-up time was 27.8 ± 13.0 months.

Coronal alignment: The mean mLDFA was 89.9° ± 6.1 preoperatively and 90.1° ± 3.5 postoperatively (*p* = 0.9). For cases without initial deformity, the mean postoperative mLDFA was 89.2° ± 3.2 and was not statistically different from the contralateral side at 88° ± 2.3 (*p* = 0.15).

A subgroup of 10 patients with preoperative angular deformity in the frontal plane was analyzed. The mean postoperative mLDFA showed significant improvement from 95.7° ± 5 preoperatively to 91.5° ± 3.4 postoperatively (*p* < 0.01).

Knee ROM in the femoral group was considered satisfactory in 28 out of 30 cases. Mean flexion was unaltered, 125° ± 25 vs. 128° ± 19 (*p* = 0.14). Two patients (gunshot wound and open floating knee injury) with limited preoperative ROM retained similar values after the surgery.

Postoperative complications were classified according to Paley and severity according to Black et al. [18,19].

There were 14/34 problems managed non-operatively:

- Eight hematomas at the osteotomy site that resolved spontaneously (severity I).
- Six lengthening delays relative to preoperative planning, which were offset by an increase in the number of daily distraction sessions (severity I).

**Figure 3:**
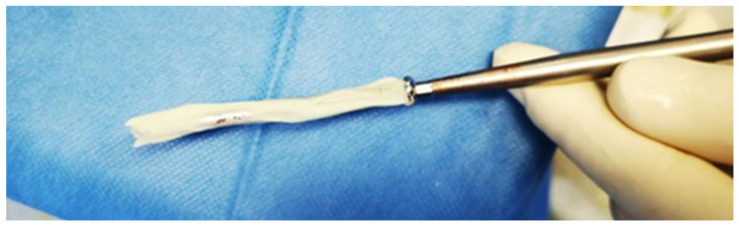
Revision of a migrating locking screw with bone cement coating. Coat the screw with regular bone cement, wait for 3–4 min before pushing and screwing it back into place. The tolerance between the screw Ø4.5 mm, the drilled hole Ø4.5 mm, and hole in the nail Ø4.6 mm ensures that no excessive cement remains within the bone. The cement acts as tight press-fitted mantle only. Most cement remains outside the lateral cortex and can be easily removed.

There were 14/34 obstacles managed operatively:

- Six cases of locking screw migration, mostly occurring during the distraction phase, and revised with cemented screws (Figure 3, severity II).
- Two cases of subcutaneous receiver removal before nail extraction because of discomfort (severity II).
- One percutaneous hamstring tenotomy to treat a persistent 50° knee flexion contracture in a patient with a 50 mm LLD. At the latest follow-up and after an intensive rehabilitation program, the flexion contracture had resolved (severity II).
- Five patients had delayed union among the femoral lengthening cases. Three of them had cancellous bone graft harvested from the anterior iliac crest; a short lateral approach was used to insert the graft after decortication of the site. This procedure was performed on average 14 months after implantation. The device had to be removed in one patient (nail breakage and malunion); treatment consisted of reaming and a trauma nail. Bone healing was achieved in all cases at the latest follow-up (severity II).
- One failure of the lengthening nail that was replaced 35 days after implantation with revision osteotomy. Lengthening proceeded satisfactorily thereafter with a final LLD of 7 mm (severity IIIA).

There were no intraoperative and 2/34 postoperative complications affecting the long-term outcome:

- One acute infection in a 54-year-old female patient with a 70 mm LLD associated with significant femur deformity and treated during childhood with monolateral external fixator. She was reoperated on postoperative day 10 with debridement and antibiotic therapy. Later on, deep vein thrombosis was found and treated with anticoagulant therapy. At the latest follow-up, the post lengthening LLD was 40 mm (severity IIIB).
- One case of significant stiffness in a 27-year-old patient with an 80 mm LLD resulting from a gunshot injury with a flexion deficit. The patient underwent Judet’s quadricepsplasty twice. The first resulted in a thrombosis of the femoral vascular graft and the second in an infection warranting surgical debridement and IV antibiotics. The limb was painful and non-functional at the last follow-up (0/0/20° knee ROM) (severity IIIB).

Thus, in 34 segments we report 8 + 6=14 severity grade I, 6 + 2 + 1 + 5 + 1 = 15 severity grade II, 1 severity grade IIIA and 2 severity grade IIIB complications, including 26 unplanned surgeries.

## 4. Discussion

Lengthening with the motorized Fitbone^®^ nail is an acceptable and predictable solution to trauma-related LLD that can also be associated with axial and rotational deformity. The presented results are consistent with previous publications [7,11,20,21,22,23].

The percentage of patients who achieved the lengthening goals defined during preoperative planning (45%) was lower than we had initially hoped for. Most of the cases where the targets were not achieved involved the largest LLD associated with complex deformities. The cases with a residual LLD of more than 1 cm at the last follow-up included several non-compliant patients who decided not to proceed until the end of the process of lengthening, one deep infection, and an equinus deformity after 40 mm tibial lengthening.

These findings underline the importance of patient selection. Careful preoperative evaluation of the motivation and the capacity of the patient to comply with the extensive and restrictive protocol (with daily lengthening sessions over several weeks, close follow-up during the distraction phase, intensive rehabilitation program, etc.) is key to increase the likelihood of good functional outcomes and minimize complications.

Any delays in the lengthening schedule were addressed with an increase in daily distraction sessions. These were likely caused by early bone consolidation, unauthorized weight-bearing, or non-compliance with the lengthening protocol.

The standard deviation of the mean femoral lengthening healing index was relatively wide (84.6 ± 62.5) due to the presence of five delayed/insufficient consolidations that were surgically managed on average at 14 months after the implantation of the nail. All five bone segments were consolidated without affecting the long-term outcome. The healing index was measured independently. It is relatively high compared with the existing literature. The healing may have been negatively affected by previous damage to the fractured bone segment, its blood supply and soft tissue conditions. Moreover, some patients were active smokers who did not cease smoking despite our recommendation.

Thus, in 34 segments we report 41% severity grade I, 41% severity grade II, 2% severity grade IIIA and 5% severity grade IIIB complications [19,24]. There were more low-grade complications in this study compared to those found in the literature and similar high-grade complications rate. In comparison, Frost in their review of the literature found 11% of complication severity grade I, 15% grade II, 5% grade IIIA and 3% grade IIIB [20]. This may be due to the low-grade complications, such as transient postoperative hematoma, that are seldom reported in the literature. None of our patients had a redon drain inserted during surgery.

We report a total of 26 unplanned surgeries in 11/34 (32%) of the segments. This high rate requires preoperative information of and consent to possible unplanned reoperation. Some of these reoperations were due to device-related complications. However, besides the recently reported adverse events of the latest generation of magnetically controlled limb lengthening nails, i.e., STRYDE, both FITBONE^®^ and titanium PRECICE^®^ have proven their clinical reliability and safety for many years [24,25,26,27,28,29]. Thaller et al. performed 241 FITBONE^®^ surgeries from 1999 until 2009 and imply that corrosion and osteolysis may also apply to a “number of FITBONE” [30]. Consequently, even though it was not the primary objective of the present study, we evaluated the latest radiographs and found no osteolysis and no periosteal reaction at the telescoping junction in our prospective cohort of 34 patients that were operated from 2010 until 2019. Moreover, we did not observe a significant amount of corrosion on the retrieved FITBONE^®^ nails, which were returned to Wittenstein intens (GmbH, Ingersheim, Germany) as part of the company’s routine quality control.

The relatively high reoperation rate in our series may partially be explained by the fact that multiple of our cases were polytrauma patients with comorbidities that could lead to complications, such as complex osseous injuries (deformity, osteoporosis due to limited weight bearing, non-union, osteomyelitis), the presence of fixation devices (broken implant, difficult extraction), as well as musculoskeletal (joint stiffness, muscle atrophy) and cutaneous disorders (scarring after open fracture, skin graft, scarring from prior surgery) [31,32]. Multidisciplinary management by the surgeon, rehabilitation specialists, infectious diseases specialist, and paramedical team is needed in such cases. We encountered no fractures of the regenerated bone or any failure due to the intramedullary nail having weakened the cortical bone.

Besides restoring limb length, the majority of associated angular and rotational deformities can also be managed using intramedullary lengthening nails. In this regard, the finding of the present study is in line with the existing literature [12,13,16,20,32,33,34,35,36]. However, in comparison with external fixators allowing gradual correction, correction of these deformities with intramedullary devices limits the postoperative options for correcting the mechanical axes of the limb. Therefore, a high degree of planning and intraoperative accuracy are essential [13,33,34,35,36]. The application of blocking screws is often needed to achieve and maintain the pre-operative plan. Sharp and rigid reamers can help to overcome potential posttraumatic challenges such as an obliterated intramedullary canal and thus help to restore limb alignment. We used this technique to position the nail in coronal deformity cases and, in our opinion, it should be more widely indicated. Indeed, it improves construct stability and nail alignment, especially in the distal femoral metaphysis. Furthermore, less stress on the locking screws theoretically reduces the risk of locking screw migration/pull-out. There were six cases of locking screw migration, which was the most common cause of revision surgery in our cohort. Other authors also report this complication [18,20]. The lack of screw threads in the far cortex and in the cancellous bone in the distal femoral metaphysis is likely to contribute to this phenomenon.

This study has several limitations. First, the studied cohort was relatively small (34 patients) and heterogenous in the causes of posttraumatic LLD. Secondly, deformity correction was only assessed for varus deformities of the femur. Thirdly, this study neither assessed patient satisfaction nor daily, physical and social activities.

## 5. Conclusions

In conclusion, bone lengthening with a motorized intramedullary nail system for posttraumatic LLD is a reliable treatment modality. The risk of serious complications and sequelae is relatively low in compliant and motivated patients. Limb length and axes can be restored with proper preoperative planning and meticulous surgery.

## Figures and Tables

**Table 1:** Anatomical site of the injuries causing posttraumatic limb length discrepancies.

		*N* = 34
Immature skeleton	Femur	10
	Tibia	2
Mature skeleton	Femur	
	Proximal	3
	Shaft	10
	Distal	6
	Tibia	2
Functional LLD	Pelvis	1

**Table 2:** Demographics, injury, pre-existing complications, and outcomes: pre- and postlengthening LLD, mechanical axis, angles and complications of the 34 lengthened patients.

ID	Gender	Age	Segment			Injury	Initial Treatment	Previous Complications	LLD (mm)	LLD (mm)	MAD (mm)	mLDFA (°)	MPTA (°)	Complications
1	M	30	Femur	proximal		Trauma in childhood	dynamic hip screw	Coxa vara	45	9				Delayed union of regenerate
2	M	13	Femur	proximal		Unknown	cannulated screws	Coxa vara	40	18	27	89	80	Screw migration
3	M	28	Femur	proximal		Motocross accident	dynamic hip screw		30	1	12	83	86	Hematoma, lengthening delay
4	M	31	Femur	proximal		Skiing accident	trauma nail		30	4	4	89	88	
5	M	36	Femur	proximal		Fall from height	trauma nail	Implant failure, osteoporosis	27	4	12	88	85	
6	F	18	Femur	shaft		Fall in childhood	ESIN		35	7	19	94	89	Receiver removal (discomfort)
7	M	31	Femur	shaft		Road traffic accident	trauma nail	Pseudarthrosis	71	30	8	90	88	
8	M	42	Femur	shaft		Road traffic accident, motorcycle	trauma nail	Pseudarthrosis	30	2	20	93	86	
9	M	26	Femur	shaft		Road traffic accident	trauma nail	Pseudarthrosis	55	5	16	89	86	Hematoma
10	M	35	Femur	shaft		Work related accident	trauma nail		40	3	14	86	82	Hematoma, lengthening delay
11	M	32	Femur	shaft		Road traffic accident, motorcycle	trauma nail		40	10	1	94	96	Hematoma
12	M	27	Femur	shaft		Ballistic trauma	external fixation	Arterial bypass due to femoral ischemia, sepsis, angular deformity, knee contracture	80	7	40	96	84	Screw migration, lengthening delay, extreme knee stiffness
13	M	42	Femur	shaft		Road traffic accident	trauma nail	Angular deformity	45	8				
14	M	53	Femur	shaft		Fall from height	trauma nail	Angular deformity	25	20	8	88	92	Screw migration
15	M	19	Femur	shaft		Motocross accident	trauma nail	Angular deformity	45	11	25	91	86	
16	M	27	Femur	shaft		Trauma in childhood	tibial traction	Angular deformity	50	6	13	95	95	
17	F	27	Femur	shaft		Road traffic accident	trauma nail		30	5	8	89	85	
18	F	26	Femur	shaft		Road traffic accident, motorcycle	ESIN	Angular deformity	30	9	30	95	87	
19	M	34	Femur	distal	open	Road traffic accident	trauma nail	Pseudarthrosis	24	3	6	86	86	Delayed union of regenerate
20	M	28	Femur	distal	open	Road traffic accident, motorcycle	plate	Knee contracture	37	6	4	88	87	Screw migration
21	M	27	Femur	distal	open	Ballistic trauma	external fixation	Septic pseudarthrosis treated with induced membrane technique, plate failure, Judet’s quadricepsplasty, angular deformity	50	7	18	95	90	Screw migration, lengthening delay, implant failure
22	M	22	Femur	distal		Trauma in childhood	cast	Fracture after lengthening with external fixator, angular deformity	55	1				Delayed union of regenerate
23	F	54	Femur	distal		Trauma in childhood	cast	Sepsis during lengthening with external fixator, angular deformity.	70	40	19	86	80	Lengthening delay, sepsis
24	M	17	Femur	distal	open	Road traffic accident, motorcycle	trauma nail		25	2	8	89	84	
25	M	17	Femur	distal		Trauma in childhood	cannulated screws		40	17	17	89	84	
26	M	14	Femur	distal	open	Trauma in childhood	Kirschner wires	Arterial bypass, sciatic palsy, sepsis, skin graft	50	14	15	91	95	Knee contracture
27	M	39	Femur	distal		Road traffic accident, motorcycle	plate		35	9	14	90	86	Hematoma, delayed union of regenerate
28	M	39	Femur	distal	open	Road traffic accident, motorcycle	plate	Angular deformity	55	1	26	95	86	Delayed union of regenerate, screw migration
29	F	26	Femur	distal		Defenestration	unkown	Angular deformity	100	2				Hematoma
30	F	26	Pelvis	Tile type C		Riding accident	plate + screws	Loss of reduction	22	1				Hematoma, lengthening delay, receiver removal (discomfort)
31	F	34	Tibia	distal	open	Road traffic accident, motorcycle	external fixation antero-medial plate		30	4	3	86	86	Hematoma
32	F	14	Tibia	distal	open	Trauma in childhood	Kirschner wires	Angular deformity	29	7	10	86	89	
33	M	24	Tibia	shaft	open	Road traffic accident, pedestrian	trauma nail		45	19	19	84	93	
34	F	17	Tibia	shaft	open	Road traffic accident, pedestrian	trauma nail	Angular deformity, skin graft	25	5	15	88	83	

M: male, F: female, ESIN: elastic stable intramedullary nailing, LLD: limb length discrepancy, MAD: mechanical axis deviation, mLDFA: mechanical lateral distal femoral angle, MPTA: medial proximal tibial angle.

## Data Availability

The data presented in this study are available on request from the corresponding author. The data are not publicly available because the patients did not provide their written consent. If data are shared and used in other non-profit publications, this paper must be cited.
